# Supplementary material for: Multiscale criticality measures as general-purpose gauges of proper brain function
Source: Sci Rep. 2021 Jul 14;11:14441. doi: 10.1038/s41598-021-93880-8 (PMC8280148; doi:10.1038/s41598-021-93880-8)
Supplement: Supplementary file 1 — Supplementary Tables. [file 41598_2021_93880_MOESM1_ESM.pdf]

SUPPLEMENTARY INFORMATION for Multiscale criticality measures as general-purpose gauges of proper brain function

Tomer Fekete, Hermann Hinrichs, Jacobo Diego Sitt, Hans-Jochen Heinze, Oren Shriki.

Group/measure	a_σ	b_σ	a_α	b_α	a_τ
Control	-0.025±0.032	1.4±0.053	0.025±0.003	-1.3±0.021	0.019±0.013
EMC	0.062±0.0096	1.3±0.041	0.028±0.0024	-1.4±0.014	0.036±0.0051
MC	0.083±0.0046	1.4±0.034	0.028±0.0011	-1.4±0.012	0.03±0.0041
VS	0.11±0.0054	1.3±0.031	0.029±0.0011	-1.4±0.012	0.043±0.0035
Group/measure	$b_{\sigma,\alpha}$	$a_{\sigma,\tau}$	$b_{\sigma,\tau}$	$a_{\sigma,\gamma}$	$b_{\sigma,\gamma}$
Control	-1.8±0.14	0.68±0.12	-3.1±0.16	0.59±0.16	-2.2±0.21
EMC	-1.7±0.085	0.44±0.057	-2.6±0.061	0.38±0.085	-1.7±0.087
MC	-1.8±0.025	0.3±0.052	-2.4±0.071	0.23±0.068	-1.5±0.09
VS	-1.7±0.019	0.42±0.036	-2.6±0.049	0.3±0.054	-1.5±0.069
Group/measure	$a_{\alpha,\delta}$	$b_{\alpha,\delta}$	$a_{\tau,\gamma}$	$b_{\tau,\gamma}$	$a_{\tau,\delta}$
Control	-3.7±0.54	-6.6±0.59	0.85±0.13	0.43±0.29	-0.77±0.45
EMC	-2.5±0.23	-5.4±0.3	1.3±0.25	1.5±0.52	-2.2±0.45
MC	-1.9±0.11	-4.5±0.14	0.93±0.11	0.66±0.23	-0.63±0.21
VS	-1.6±0.11	-4±0.15	0.76±0.12	0.37±0.25	-0.85±0.14
b_τ	a_γ	b_γ	a_δ	b_δ	$a_{\sigma,\alpha}$
-2.2±0.026	0.022±0.0083	-1.4±0.033	-0.077±0.0041	-1.8±0.038	0.34±0.093
-2.1±0.023	0.031±0.0041	-1.2±0.034	-0.064±0.0042	-1.9±0.034	0.28±0.064
-2.1±0.016	0.023±0.0042	-1.3±0.021	-0.054±0.0028	-1.8±0.028	0.34±0.016
-2.1±0.013	0.026±0.0048	-1.2±0.022	-0.045±0.0029	-1.8±0.025	0.26±0.013
$a_{\sigma,\delta}$	$b_{\sigma,\delta}$	$a_{\alpha,\tau}$	$b_{\alpha,\tau}$	$a_{\alpha,\gamma}$	$b_{\alpha,\gamma}$
-0.73±0.26	-0.89±0.34	0.014±0.69	-2.1±0.88	0.61±0.46	-0.58±0.61
-0.52±0.22	-1.3±0.27	1.2±0.13	-0.45±0.19	1.3±0.24	0.46±0.36
-0.67±0.055	-0.96±0.087	0.88±0.16	-0.86±0.21	0.82±0.19	-0.17±0.25
-0.41±0.036	-1.3±0.051	1.5±0.15	0.07±0.2	1.2±0.27	0.48±0.36
$b_{\tau,\delta}$	$a_{\gamma,\delta}$	$b_{\gamma,\delta}$	κ	κ_{gen}	$\alpha_{dropoff}$
-3.4±1	-1.1±0.45	-3.3±0.69	1±0.0025	0.97±0.002	2.1e+02±26
-6.7±0.95	-1.3±0.2	-3.5±0.29	1±0.0019	0.98±0.0012	2.8e+02±22
-3.1±0.47	-0.68±0.17	-2.7±0.24	1±0.0016	0.98±0.001	2.8e+02±14
-3.6±0.3	-0.36±0.11	-2.2±0.15	1±0.0014	0.98±0.0011	2.8e+02±12

Supplementary Table 1: *Group averages and standard errors of the 33 MsCr measures used in this study derived from the DOC dataset*

Group/measure	a_{σ}	b_{σ}	a_{α}	b_{α}	a_{τ}
Control	0.03±0.0032	0.58±0.017	0.036±0.0016	-1.6±0.015	0.063±0.0058
MCI	0.013±0.0056	0.77±0.044	0.027±0.0016	-1.5±0.017	0.025±0.0056
Group/measure	$b_{\sigma,\alpha}$	$a_{\sigma,\tau}$	$b_{\sigma,\tau}$	$a_{\sigma,\gamma}$	$b_{\sigma,\gamma}$
Control	-2±0.029	1.9±0.11	-3.5±0.052	2.1±0.15	-2.8±0.086
MCI	-1.7±0.046	1.2±0.088	-3.2±0.053	1.1±0.14	-2.3±0.11
Group/measure	$a_{\alpha,\delta}$	$b_{\alpha,\delta}$	$a_{\tau,\gamma}$	$b_{\tau,\gamma}$	$a_{\tau,\delta}$
Control	-2±0.11	-4.9±0.17	1.2±0.051	1.4±0.13	-0.72±0.037
MCI	-2.3±0.13	-5±0.22	1.1±0.069	0.95±0.16	-0.23±0.085
b_{τ}	a_{γ}	b_{γ}	a_{δ}	b_{δ}	$a_{\sigma,\alpha}$
-2.4±0.02	0.091±0.0045	-1.5±0.024	-0.071±0.0031	-1.6±0.014	0.71±0.045
-2.4±0.016	0.048±0.0086	-1.6±0.025	-0.062±0.0026	-1.5±0.023	0.28±0.055
$a_{\sigma,\delta}$	$b_{\sigma,\delta}$	$a_{\alpha,\tau}$	$b_{\alpha,\tau}$	$a_{\alpha,\gamma}$	$b_{\alpha,\gamma}$
-1.2±0.13	-0.9±0.076	1.6±0.11	0.22±0.17	2.5±0.11	2.6±0.19
-0.33±0.13	-1.3±0.087	0.58±0.19	-1.4±0.27	1.7±0.35	1.2±0.52
$b_{\tau,\delta}$	$a_{\gamma,\delta}$	$b_{\gamma,\delta}$	κ	κ_{gen}	$\alpha_{dropoff}$
-3.3±0.094	-0.72±0.042	-2.7±0.073	0.98±0.00077	0.99±0.0011	43±3.5
-2.1±0.22	-0.49±0.085	-2.2±0.15	0.99±0.002	0.98±0.0013	61±7.6

Supplementary Table 2: *Group averages and standard errors of the 33 MsCr measures used in this study derived from the MCI dataset*

Group/measure	a_{σ}	b_{σ}	a_{α}	b_{α}	a_{τ}
Control	0.013±0.0055	1.1±0.068	0.0093±0.00043	-1.3±0.009	0.027±0.0016
Schiz	0.06±0.041	1.4±0.22	0.011±0.00065	-1.3±0.015	0.016±0.0022
Group/measure	$b_{\sigma,\alpha}$	$a_{\sigma,\tau}$	$b_{\sigma,\tau}$	$a_{\sigma,\gamma}$	$b_{\sigma,\gamma}$
Control	-1.4±0.02	0.34±0.047	-2.6±0.05	0.28±0.097	-1.6±0.1
Schiz	-1.3±0.028	0.2±0.066	-2.5±0.067	0.2±0.13	-1.6±0.13
Group/measure	$a_{\alpha,\delta}$	$b_{\alpha,\delta}$	$a_{\tau,\gamma}$	$b_{\tau,\gamma}$	$a_{\tau,\delta}$
Control	-3.5±0.15	-6.2±0.2	1.6±0.13	2.3±0.28	-0.99±0.074
Schiz	-2.8±0.21	-5.1±0.27	1.2±0.16	1.4±0.36	-0.83±0.17
b_{τ}	a_{γ}	b_{γ}	a_{δ}	b_{δ}	$a_{\sigma,\alpha}$
-2.3±0.018	0.042±0.0023	-1.3±0.024	-0.035±0.0011	-1.7±0.012	0.082±0.016
-2.3±0.018	0.027±0.003	-1.4±0.028	-0.032±0.0017	-1.6±0.02	0.065±0.024
$a_{\sigma,\delta}$	$b_{\sigma,\delta}$	$a_{\alpha,\tau}$	$b_{\alpha,\tau}$	$a_{\alpha,\gamma}$	$b_{\alpha,\gamma}$
-0.2±0.061	-1.5±0.063	2.9±0.18	1.5±0.23	4.5±0.4	4.5±0.53
-0.14±0.08	-1.5±0.083	1.5±0.18	-0.29±0.23	2.4±0.37	1.7±0.46
$b_{\tau,\delta}$	$a_{\gamma,\delta}$	$b_{\gamma,\delta}$	κ	κ_{gen}	$\alpha_{dropoff}$
-4±0.17	-0.54±0.038	-2.5±0.057	1±0.0027	0.97±0.00074	1.9e+02±12
-3.5±0.39	-0.48±0.085	-2.3±0.13	1±0.0025	0.96±0.0018	1.6e+02±13

Supplementary Table 3: *Group averages and standard errors of the 33 MsCr measures used in this study derived from the Schizophrenia dataset.*

Group/measure	a_{σ}	b_{σ}	a_{α}	b_{α}	a_{τ}
preictal	0.031±0.00043	0.44±0.002	0.035±0.00067	-1.8±0.0029	0.061±0.00082
interictal	0.033±0.00046	0.45±0.0023	0.037±0.003	-1.8±0.0074	0.061±0.00088
Group/measure	$b_{\sigma,\alpha}$	$a_{\sigma,\tau}$	$b_{\sigma,\tau}$	$a_{\sigma,\gamma}$	$b_{\sigma,\gamma}$
preictal	-2.2±0.0072	1.8±0.021	-3.2±0.0089	1.9±0.032	-2.3±0.013
interictal	-2.1±0.015	1.7±0.026	-3.1±0.01	1.6±0.087	-2.2±0.042
Group/measure	$a_{\alpha,\delta}$	$b_{\alpha,\delta}$	$a_{\tau,\gamma}$	$b_{\tau,\gamma}$	$a_{\tau,\delta}$
preictal	-1.1±0.017	-3.5±0.028	1.2±0.01	1.3±0.024	-0.4±0.012
interictal	-1±0.018	-3.3±0.03	1.1±0.01	1.2±0.025	-0.36±0.013
b_{τ}	a_{γ}	b_{γ}	a_{δ}	b_{δ}	$a_{\sigma,\alpha}$
-2.5±0.004	0.067±0.001	-1.6±0.0049	-0.041±0.00054	-1.5±0.0042	0.97±0.016
-2.4±0.0043	0.064±0.001	-1.5±0.0052	-0.036±0.00055	-1.5±0.0044	0.97±0.11
$a_{\sigma,\delta}$	$b_{\sigma,\delta}$	$a_{\alpha,\tau}$	$b_{\alpha,\tau}$	$a_{\alpha,\gamma}$	$b_{\alpha,\gamma}$
-1.1±0.027	-1.1±0.011	1.7±0.022	0.52±0.038	2±0.032	1.9±0.055
-0.89±0.048	-1.1±0.022	1.7±0.022	0.5±0.037	1.9±0.031	1.8±0.054
$b_{\tau,\delta}$	$a_{\gamma,\delta}$	$b_{\gamma,\delta}$	κ	κ_{gen}	$\alpha_{dropoff}$
-2.5±0.031	-0.34±0.0086	-2.1±0.016	0.96±0.00029	0.99±0.00016	27±1.2
-2.4±0.033	-0.28±0.009	-2±0.016	0.96±0.00047	0.99±0.00015	34±1.7

Supplementary Table 4: *Group averages and standard errors of the 33 MsCr measures used in this study derived from the epilepsy dataset.*

Data/measure	a_{σ}	b_{σ}	a_{α}	b_{α}	a_{τ}	b_{τ}	a_{γ}	b_{γ}	a_{δ}	b_{δ}	$a_{\sigma,\alpha}$
DOC	***					*		**	**		
MCI		**	**	**	***		**			*	***
Schiz											
Epilepsy	***	**				**		***	***		
Data/measure	$b_{\sigma,\alpha}$	$a_{\sigma,\tau}$	$b_{\sigma,\tau}$	$a_{\sigma,\gamma}$	$b_{\sigma,\gamma}$	$a_{\sigma,\delta}$	$b_{\sigma,\delta}$	$a_{\alpha,\tau}$	$b_{\alpha,\tau}$	$a_{\alpha,\gamma}$	$b_{\alpha,\gamma}$
DOC			**		*			*	*		
MCI	***	***	**	***	**	***	**	***	***		
Schiz											
Epilepsy		***	***	*	*	*					
Data/measure	$a_{\alpha,\delta}$	$b_{\alpha,\delta}$	$a_{\tau,\gamma}$	$b_{\tau,\gamma}$	$a_{\tau,\delta}$	$b_{\tau,\delta}$	$a_{\gamma,\delta}$	$b_{\gamma,\delta}$	κ	κ_{gen}	$\alpha_{dropoff}$
DOC	***	***			*	*				*	
MCI					***	***		*		**	
Schiz											
Epilepsy	***	***					***	***	***		*

***Supplementary Table 5**: Significance of MsCr features across all data sets employing parametric statistics. *denotes p<0.01 **denotes p<0.001 ***denotes p<0.0001*

Data/measure	a_{σ}	b_{σ}	a_{α}	b_{α}	a_{τ}	b_{τ}	a_{γ}	b_{γ}	a_{δ}	b_{δ}	$a_{\sigma,\alpha}$
DOC	***					*		**	***		*
MCI		*	*	*	**						**
Schiz		*			**		*			***	
Epilepsy	***	***		**		***		***	***		***
Data/measure	$b_{\sigma,\alpha}$	$a_{\sigma,\tau}$	$b_{\sigma,\tau}$	$a_{\sigma,\gamma}$	$b_{\sigma,\gamma}$	$a_{\sigma,\delta}$	$b_{\sigma,\delta}$	$a_{\alpha,\tau}$	$b_{\alpha,\tau}$	$a_{\alpha,\gamma}$	$b_{\alpha,\gamma}$
DOC			*		*	*		*	*		
MCI	**	**	*	**	*	**		*	*		
Schiz								***	***	**	**
Epilepsy	***	***	***	***	***	***	***				
Data/measure	$a_{\alpha,\delta}$	$b_{\alpha,\delta}$	$a_{\tau,\gamma}$	$b_{\tau,\gamma}$	$a_{\tau,\delta}$	$b_{\tau,\delta}$	$a_{\gamma,\delta}$	$b_{\gamma,\delta}$	κ	κ_{gen}	$\alpha_{dropoff}$
DOC	***	***			*	*	*	*		*	
MCI					*	*				*	
Schiz										*	
Epilepsy	***	***	*		***	***	***	***	***		***

Supplementary Table 6: *Significance of MsCr features across all data sets employing non-parametric statistics. *denotes p<0.01 **denotes p<0.001 ***denotes p<0.0001*
